# Comparison of two multiband mucosectomy devices for endoscopic resection of Barrett’s esophagus-related neoplasia

**DOI:** 10.1007/s00464-018-06655-0

**Published:** 2019-01-22

**Authors:** Durayd Alzoubaidi, David Graham, Paul Bassett, Cormac Magee, Martin Everson, Matthew Banks, Marco Novelli, Marnix Jansen, Laurence B. Lovat, Rehan Haidry

**Affiliations:** 1 Division of Surgery & Interventional Science, University College London (UCL), Charles Bell House, 43-45 Foley Street, London, W1W 7TS UK; 2 Department of Gastroenterology, University College London Hospital NHS Foundation Trust, London, UK; 3 Department of Pathology, University College London Hospital NHS Foundation Trust, London, UK; 4 Statsconsultancy Ltd, 40 Longwood Lane, Amersham, HP7 9EN UK

**Keywords:** Barrett’s esophagus (BE), Esophageal adenocarcinoma (EAC), Endoscopic mucosal resection, Multiband mucosectomy (MBM)

## Abstract

**Background:**

Esophageal adenocarcinoma carries a poor prognosis and therefore treatment of early neoplasia arising in the precursor condition Barrett’s esophagus (BE) is desirable. Visible lesions arising in BE need endoscopic mucosal resection for accurate staging and removal. Resection modalities include a cap-based system with snare and custom-made multiband mucosectomy (MBM) devices (Duette, Cook Medical Ltd). A new MBM device has recently become available (Captivator, Boston Scientific Ltd).

**Objectives:**

A retrospective pilot study to compare the efficacy, safety, specimen size and histology of endoscopic mucosal resection (EMR) specimens resected with two MBM devices (Cook Duette and Boston Captivator) in treatment naive patients undergoing endoscopic therapy for BE neoplasia.

**Methods:**

Consecutive EMR procedures carried out by a single experienced endoscopist were analysed. All visible lesions were marked and resected using one of the two MBM devices. All resected specimens were analysed by the same two experienced pathologists. The resected specimens in both groups were analysed for maximum diameter, minimum diameter, surface area and depth.

**Results:**

Twenty consecutive patients were analysed (18M + 2F; mean age 74) in the Duette group and 20 (17M + 3F; mean age 72) in the Captivator group. A total of 58 specimens were resected in the Duette and 63 in the Captivator group. Min diameter, max diameter, surface area and depth of the ER specimens resected by the Captivator device were significantly larger than that by the Duette device [min diameter 9.89 mm vs 9.07 mm (*p* = 0.019); max diameter: 13.54 mm vs 12.38 mm (*p* = 0.024); surface area: 135.40 mm^2^ vs 113.89 mm^2^ (*p* = 0.005); depth 3.71 mm vs 2.89 (*p* = 0.001)].

**Conclusions:**

These two MBM devices showed equivalent efficacy and safety outcomes, but the EMR Captivator device resected specimens with a larger area in the esophagus when compared with the Duette device. A possible advantage of this is in situations where en bloc resections with fewer EMRs are desirable for larger lesions.

Barrett’s esophagus (BE) is a precancerous condition that predisposes to esophageal adenocarcinoma (EAC) and is characterised by a change of normal squamous epithelium lining the esophagus to metaplastic columnar epithelium due to chronic acid reflux [1]. The incidence of EAC in Western countries has increased in recent years and despite advances in surgical and oncological interventions, long-term survival remains poor. Surgical management of early esophageal neoplasia carries significant mortality rates [2–4]. In recent years there have been significant developments in minimally invasive endoscopic eradication therapy (EET) of BE neoplasia with high eradication rates and a good safety profile; therefore, there has been more emphasis on targeting patients at an earlier stage which can be amenable to EET that can improve patient outcomes. Endoscopic therapy of dysplastic BE and adenocarcinoma has been recommended by various major societal guidelines [5, 6].

Current consensus is that visible lesions arising in BE are removed by endoscopic resection (ER) as they may harbour the most advanced histological stage. Accurate staging with endoscopic mucosal resection (EMR) is a key step in the treatment of early neoplasia as it allows accurate risk stratification of patients. Resection specimens provide information on depth of mucosal or submucosal invasion and presence or absence of lympho-vascular invasion, which subsequently would allow appropriate modalities of further treatment to be offered [7]. ER is effective and safe in selected patients with early BE neoplasia with significantly high (up to 94%) long-term complete remission rates and low major complication rates [8, 9]. The endoscopic management of early BE neoplasia is the preferred treatment modality as surgical options carry a much higher complication rate [2, 3, 10].

Historically, the cap-based system with snare (Olympus Ltd.), initially described in Japan by Inoue et al. [11], was used. This is an ER modality that uses a transparent cap placed distally at the tip of the endoscope. The cap contains a distal internal ridge, allowing the placement of a snare in the cap prior to resection. The submucosal space is initially injected for lifting and subsequently the mucosa is suctioned into the cap in order to create a pseudopolyp. The pseudopolyp is then resected by closing the snare at the base of the pseudopolyp and applying electrocautery. This technique is a rather complicated process for the less experienced endoscopists, as it requires submucosal lifting and placing the snare at the distal ridge of the transparent cap prior to resection. This technique also results in prolonged procedure time in cases requiring multiple resections [12].

Cap EMR has a role in select cases but has now been widely replaced by the custom-made multiband mucosectomy (MBM) devices that utilises a transparent cap placed distally at the tip of the endoscope. The cap carries multiple pre-loaded rubber bands that are connected to a hand-operated controller fixed at the proximal aspect of the accessory channel. The neoplastic mucosa is suctioned into the transparent cap, followed by release of a band by the controller, resulting in the creation of a pseudopolyp. The contraction of the rubber band at the base of the pseudopolyp is only adequate to withhold the mucosa but not the underlying muscularis propria, hence the injection of the submucosal space is not routinely required. A snare is passed through the accessory channel of the endoscope and then is placed and closed at the base of the pseudopolyp, beneath the band. The pseudopolyp is then resected using electrocautery [13]. This is an easier method and the learning curve for MBM is shorter compared with that of ER cap as it combines the commonly known techniques of variceal band ligation and polypectomy [12].

The most commonly utilised MBM device is the Duette® Multi-Band Mucosectomy device (Cook Medical, Limerick, Ireland) [14]. It is a modified version of the variceal band ligator that allows the passage of a snare into the working channel of the endoscope (Fig. 1) [15]. A new MBM device has been launched (Captivator, Boston Scientific Ltd). This device also consists of a cap placed at the distal end of the scope with a controller placed at the proximal aspect of the working channel. The cap carries six rubber bands that are placed at the proximal aspect of the cap allowing 360° peripheral viewing through the transparent cap without obstructions by the ligator bands (Fig. 2). An in vitro assessment of the performance of the new EMR Captivator device by Schölvinck et al. showed that the new MBM device potentially allows better visualisation through the cap and easier passage of accessories through the scope with significantly better suction power [16].

**Fig. 1:**
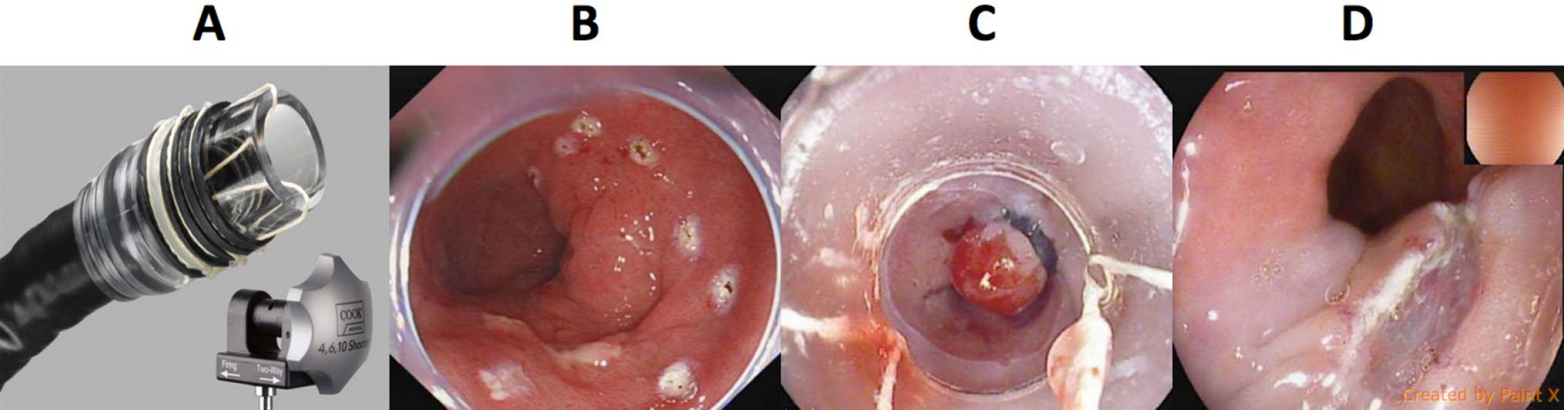
Duette EMR device. The single-use Duette MBM device consists of a transparent cap with six rubber bands and a control handle (**A**). The transparent cap is mounted at the tip of the endoscope. With a trigger cord, the six rubber bands on the outside of the transparent cap are connected to the control handle at the proximal end of the accessory channel. Without prior submucosal injection for lifting, the neoplastic lesion is delineated with the tip of the hot snare (**B**) and suctioned into the cap until a complete red out occurred on the screen due to the entire cap being filled with mucosa and then a pseudopolyp is created by releasing a rubber band (**C**). The pseudopolyp is then resected (**D**) by placing and tightening the snare beneath the rubber band

**Fig. 2:**
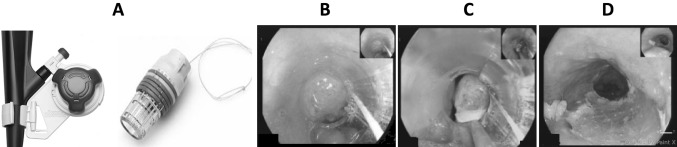
Captivator EMR device. The Captivator™ EMR device is a single-use device. The device includes the Captivator™ EMR Band Ligator mounted at the proximal aspect of the accessory channel and a banding cap device placed at the distal end of the scope for creation of pseudopolyps (**A**). A pseudopolyp is created by suctioning the neoplastic mucosa into the cap (**B**) until a complete red out occurred on the screen due to the entire cap being filled with mucosa and then a band is deployed using a proximally attached band ligator (**C**). A snare is then passed through the accessory channel of the scope, placed over the pseudopolyp and then closed beneath the rubber band (**C**), the pseudopolyp is resected (**D**) in conjunction with coagulation current. The device can be used for up to six resections

## Objectives

The primary objectives of this study were to assess the efficacy (defined by successful resection of all the delineated areas in one single session) and safety of the two MBM devices (Cook Duette and Boston Captivator) in treatment of naïve patients with BE neoplasia undergoing EMR.

Secondary objectives included retrospective comparison of the size of the resected EMR specimens by the two MBM devices in consecutive patients with BE neoplasia. Minimum diameter, maximum diameter, surface area and depth (defined as microscopically measured thickness) of the resected EMR specimens were compared to identify if either of the devices is capable of resecting larger EMR specimens. Final histology of EMR specimens obtained by the two MBM devices was also compared.

## Materials and methods

### Patient selection and inclusion criteria

A retrospective study looking at treatment naive patients (defined as those with no prior endotherapy and radiotherapy) with BE neoplasia undergoing EMR from March 2015 to October 2017. Consecutive patients treated by the Cook Duette or the Boston Captivator device in a high-volume tertiary referral centre were analysed. Patients aged 18–90 years with a visible lesion detected on white light endoscopy, narrow band imaging or optical enhancement, confirmed on recent endoscopy and deemed suitable for ER were included. Written informed consent was obtained from all patients prior to the procedure.

### Exclusion criteria

Patients with previous esophageal EET, including EMR/endoscopic submucosal dissection (ESD), radio frequency ablation, cryoablative therapy, laser treatment, photodynamic therapy, argon plasma coagulation or radiotherapy were excluded from the study. In addition, patients with esophageal stenosis (preventing the passage of a gastroscope), esophageal varices and coagulopathy were also excluded from this study.

IRB approval was not required as this project was a retrospective audit of routine clinical care and deemed exempt as per UK guidelines on clinical audit [17].

### Endoscopic procedure

All ERs were performed by a single experienced senior endoscopist with extensive experience in EMR in the esophagus using both the Duette and the Captivator devices. The same therapeutic gastroscope with a 3.2-mm working channel was used.

At the time of endoscopy, the distance of the visible lesions (centimetre) from the incisors was recorded in addition to the location and estimated size of the lesion (millimetre). Lesions were classified according to the Paris classification [18]. The length of the BE segment was also defined as per Prague Classification [19]. Visible lesions were delineated (Fig. 1) with the tip of the device snare (ERBE VIO 300D, Forced Coag, Effect 2, 40 W). After delineation, lesions were resected using one of the two MBM devices: Duette or Boston Captivator (Figs. 1, 2). The decision on which device was used was non-randomised and not controlled for in the study and device selection was done at the outset of each case at the endoscopist’s discretion.

Immediately after the resection in both groups, the snare was retracted; the resected specimen was pushed into the stomach and the resection base was inspected. Subsequent resections were performed (if necessary) in the same way to cover all marked areas, with only a small overlap between adjacent resections to prevent residual tissue bridges. After completion of resection of the delineated area, the resection base was carefully re-inspected to ensure that all delineation markings have been removed.

**Fig. 3:**
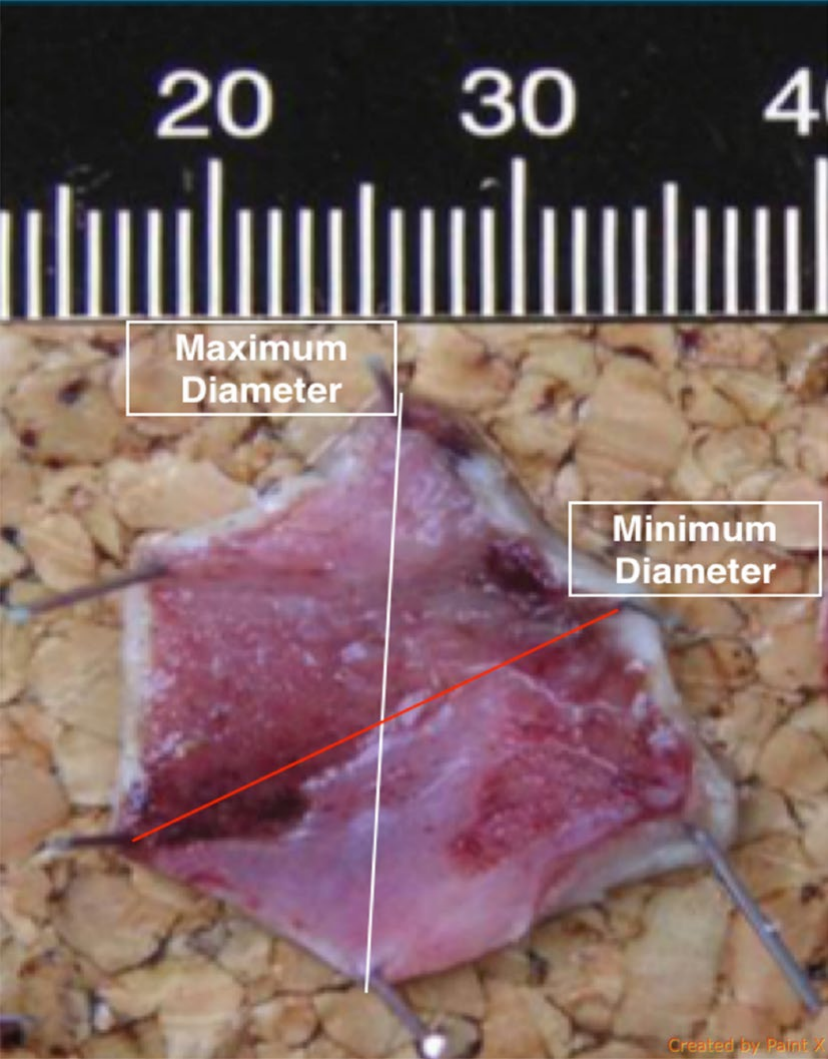
EMR specimen post resection. Pinned down on cork board, showing the maximum diameter and the minimum diameter. These measurements were done macroscopically by the GI pathologist

Identical diathermy setting (ERBE VIO 300D, Forced Coag, Effect 2, 40 W) and suction pressures (100 kPa) were used in all resections. Submucosal injection and lifting of the mucosa was not used in any of the cases. All resected specimens were successfully retrieved from the stomach using a Roth Net® (US endoscopy, a subsidiary of STERIS corporation). All specimens were pinned down to cork board (Fig. 3) by the same endoscopy nurses and preserved in identical volumes of formalin for histological evaluation by the same two experienced senior GI pathologists.

The endoscopist and the endoscopy nurses were not blinded to the type of device used but the GI pathologists (performing the measurements on the specimens) were not aware of the devices used for the mucosal resection.

### Preparation of histological specimens

EMR specimens were placed in formalin after fixation to a non-absorbent cork board (Fig. 3), then sectioned in 2-mm slices and embedded in paraffin, after which 4-µm thick slices were cut, placed on glass slides and stained with haematoxylin and eosin. The dimensions of resected EMR specimens were measured macroscopically by the pathologist prior to sectioning. These included the maximum diameter and the minimum diameter (Fig. 3). The depth (defined as the microscopically measure thickness) of each EMR specimen including submucosal invasion was measured microscopically by the pathologist. These measurements were provided on the histology report. Grading of intraepithelial neoplasia was in concordance with the Vienna classification [20]. The surface area (mm^2^) of each specimen was then approximated by multiplying the minimum diameter (millimetre) by the maximum diameter (millimetre) for each specimen.

### Statistical analysis

Statistical analysis was performed using the SPSS Statistics software (Version 25). Quantitative variables were expressed as means (± SD) and qualitative variables were presented as percentages. The mean value in the two groups (Duette and Captivator) was compared using Student’s *t* test. Fisher’s exact test used to compare R0 and R1 between the two groups.

## Results

The study included 20 patients in each group (Duette: 18M + 2F; mean age 74 years; Captivator: 17M + 3F; mean age 72 years) with a mean length of BE of C4M6 in the Duette and C3M5 in the Captivator group, *p* = NS (Table 1). The endoscopically estimated mean lesion diameter was 12 mm in the Duette and 15 mm in the Captivator group (*p* = 0.22). This estimate was carried out prior to resection by the endoscopist. Successful resection was achieved in 100% of the cases with a total of 58 specimens resected in the Duette and 63 in the captivator group. The mean number of EMRs performed per delineated lesion was 2.6 in the Duette and 2.8 in the Captivator group, *p* = 0.67 (Table 2).

**Table 1:** Patient demographic and mean Prague classification for the Duette and the Captivator group

	Duette	Captivator	*t* Test
Number of patients	20 (18M + 2F)	20 (17M + 3F)	
Mean age	74 years	72 years	*p* = 0.51
SD	± 9	± 10	
95% CI	70–78	67–76	
Mean Prague classification			
C	4	3	*p* = 0.76
Range	0–15	0–13	
M	6	5	*p* = 0.85
Range	1–15	1–15	

*SD* standard deviation, *95% CI* 95% confidence interval

**Table 2:** Total number of specimens, mean endoscopically estimated lesion diameter and mean number of EMR per lesion for the Duette and the Captivator group

	Duette	Captivator	*t* Test
Total number of specimens	58	63	
Mean endoscopically estimated lesion diameter (mm)	12	15	*p* = 0.22
SD	± 9	± 13	
95% CI	7–16	10–21	
Mean no. of EMR per lesion	2.6	2.8	*p* = 0.67
SD	± 1.6	± 2.1	
95% CI	1.9–3.4	1.9–3.7	

*SD* standard deviation, *95% CI* 95% confidence interval

### Histology

All lesions were described using the Paris classification prior to EMR. Paris IIa was the most common lesion seen (Table 3) in both groups [80% (16/20) in the Captivator and 75% (15/20) in the Duette group; *p* = 0.70]. Table 3 shows the Paris classification of all the lesions.

**Table 3:** Comparison of Paris classification of all the lesions in the Duette and the Captivator group (*p* = 0.70)

Paris classification	Is	Ip	IIa	IIb	IIa/IIc
Captivator	2/20 (10%)	0	**16**/**20 (80%)**	1/20 (5%)	1/20 (5%)
Duette	0	1/20 (5%)	**15**/**20 (75%)**	2/20 (10%)	2/20 (10%)

Nineteen patients in the Captivator group had EMR specimens with clear deep margin in comparison to 17 in the Duette group, *p* = 0.61 (Table 4). Fifteen of the patients in the Captivator group showed cancer on the EMR specimens in comparison to 12 in the Duette group (*p* = 0.50). Of those with cancer on EMR specimens, 50% showed submucosal involvement in the Duette group and 20% in the Captivator group, *p* = 0.13 (Table 5).

**Table 4:** Invasion of deep margin of EMR specimens with BE neoplasia in the Duette and the Captivator group

	R0	R1
Number of patients in the Captivator group	19/20 (95%)	**1**/**20 (5%)**
Number of patients in the Duette group	17/20 (85%)	**3**/**20 (15%)**
Fisher’s exact test	*p* = 0.61	

**Table 5:** Cancer cases with submucosal invasion based on the EMR specimens in the Duette and the captivator group

	Cancer	Mucosal cancer	Submucosal cancer
Number of patients in the Captivator group	**15**/**20 (75%)**	12/15 (80%)	**3**/**15 (20%)**
Number of patients in the Duette group	**12**/**20 (60%)**	6/12 (50%)	**6** /**12 (50%)**
Fisher’s exact test	***p*** = **0.50**	*p* = 0.13	

### EMR specimen size comparison

The mean minimum diameter, maximum diameter, surface area and depth of all resected specimens with the Captivator device was compared with that resected by the Duette device (Table 6). The data showed that the Captivator EMR specimens to be significantly larger than similar specimens resected with the Duette device [minimum diameter 9.89 mm vs 9.07 mm (*p* = 0.019); maximum diameter: 13.54 mm vs 12.38 mm (*p* = 0.024); surface area: 135.40 mm^2^ vs 113.89 mm^2^ (*p* = 0.005); depth 3.71 mm vs 2.89 (*p* = 0.001)].

**Table 6:** Comparing specimen size between the Duette and the Captivator group

	Duette group	Captivator group	*t* Test
Number of specimens	58	61	
Mean min diameter (mm)	9.07	9.89	*p* = 0.019
SD	± 1.99	± 1.76	
Lower 95% CI of mean	8.55	9.45	
Upper 95% CI of mean	9.59	10.33	
Mean max diameter (mm)	12.38	13.54	*p* = 0.024
SD	± 2.63	± 2.89	
Lower 95% CI of mean	11.69	12.82	
Upper 95% CI of mean	13.06	14.26	
Mean surface area (mm^2^)	113.89	135.40	*p* = 0.005
SD	± 38.75	± 42.68	
Lower 95% CI of mean	103.83	124.77	
Upper 95% CI of mean	123.95	146.02	
Mean depth (mm)	2.89	3.71	*p* = 0.001
SD	± 1.19	± 1.53	
Lower 95% CI of mean	2.58	3.33	
Upper 95% CI of mean	3.20	4.09	

*SD* standard deviation, *CI* confidence interval

### Complications

There were no reported perforations in either group. There was minor bleeding during the procedure that occurred in two (10%) patients in the Captivator group and one (5%) patient in the Duette group (*p* = NS). These were successfully treated with the tip of the hot snare, and there was no reported re-bleeding or hospitalisation. In our study, re-bleeding was only considered a relevant complication if it led to unplanned admission, endoscopic re-intervention and the need for blood transfusion. There was 1 (5%) delayed bleed at 48 h post Captivator EMR and 1 (5%) at 9 days post Duette EMR (*p* = NS). Both cases required conventional endoscopic therapy that was successful on first attempt. Both patients had an in-patient stay of 48 h post endotherapy for routine observation only. First follow-up endoscopy (3-months post EMR) showed 1 (5%) stricture in both groups (*p* = NS) requiring one endoscopic dilatation.

## Discussion

ER for visible BE neoplasia can achieve successful outcomes if diagnosed at an early stage [21–23]. Minimally invasive EET has significantly developed in the past decade and has shown improved mortality and morbidity in comparison to surgical management of early BE neoplasia [2, 3].

MBM is a widely used technique for the ER of neoplasia in the esophagus. MBM is effective in selected groups of patients [9] and it allows safe piecemeal resections in patients with BE neoplasia. Time and costs are saved compared with the cap and snare technique [24].

This study showed that the EMR specimens resected with Captivator device appear to have a larger minimum diameter, maximum diameter, surface area and depth in the esophagus when compared with the Duette device in similar treatment naive BE segments. Baseline lesion morphology and subsequent resection pathology were similar in both cohorts of examined patients. A possible clinical advantage of this is in situations where en bloc resection is wanted for larger or more extensive lesions (> 10 mm) with fewer resections per lesion. This may also have a positive impact on reducing procedure time as fewer resections may be needed for any given lesion size and shorter procedure time is known to reduce the total cost of treatment [12]; however our study did not formally assess the procedure time between the two groups and we do not have data to support this notion in this study. In addition, fewer resections may reduce the number of complications such as bleeding and perforation; however, our study showed no significant difference between the two groups with regards to bleeding and there were no recorded perforations. This is a potential objective for future studies on the Captivator device. Successful resection was achieved in 100% of the cases which illustrates that both devices are very effective in this respect.

Complete resection of an extensively large lesion during the first endotherapy session is desirable as subsequent strictures and fibrosis may preclude further endotherapy and resection. Also resecting larger areas at baseline endoscopy may leave less residual BE reducing the number of sessions for further endotherapy with ablation and the potential need for rescue EMR [25]. A large study by Pech et al. from 1000 consecutive patients with IMC suggested that complete removal of the whole neoplastic lesion in one session is favourable in order to reduce the risk of treatment failure [9]. This further supports the use of the Captivator device in patients with large lesions requiring complete successful resection in one session.

A previous study by Matsuzaki et al. demonstrated that larger ER specimens result in deeper resections [26]. Our study was able to show that the Captivator device resected specimens that had significantly larger microscopically measured depth in comparison to that with the Duette device; however, this did not result in higher perforation or bleeding rates, which were not significantly different, compared to that in the Duette group. The deep resection margins and radicality of neoplasia resection in our cohort of cases was not different in both the cancer and dysplasia cases. Deeper resection may be an important factor to consider in patients with suspicions of submucosal invasion at baseline. In these patients, for example, those that have significant contraindications to surgery, EET with a device with the potential of deeper resection capability may provide them with the best chance of curative endoscopic therapy. Larger and deeper EMR specimens also allow more precise evaluation of the depth of tumour penetration than any other available methods, which would allow differentiation of mucosal from submucosal tumours [25]. Large EMR specimens may be able to identify patients with submucosal invasion suitable for escalation to surgical management and therefore excluded from endoscopic therapy that may result in a less favourable long-term outcome.

In recent years, en bloc resection with ESD in large lesions have become attractive in the management of patients with BE neoplasia [27–29]. ESD is only available in expert centres with highly skilled operators. The use of the EMR Captivator device in BE neoplasia can potentially mimic this for larger lesions by acquiring larger tissue specimens and therefore in comparison to the Duette device, it may become the preferred tool for larger lesions.

Both MBM devices were shown to be equally safe and effective at resecting visible lesions in patients with BE neoplasia when performed by an experienced endoscopist in identical clinical environment. The intra-procedural acute minor bleeding episodes were considered clinically irrelevant because all were treated endoscopically during the same procedure by coagulation using the tip of the hot snare. The intra-procedural acute minor bleeding and delayed bleeding in both groups were not significantly different. The acute and delayed bleeding rates did not reflect that of recently published data [22, 23]. There were no reported perforations. Sample size was not calculated and therefore the patient numbers in both groups may have been inadequate to show statistically significant difference in complication rates between the Captivator and the Duette group. We emphasise that this was a clinical audit and feasibility analysis that may in due course support a large-scale powered RCT. In addition, the stricture rates for both groups (Captivator 5%, Duette 5%; *p* = NS) were lower than that documented in major recent studies (10–37%) [22, 23]; however, one must take into account that all procedures were performed by the same senior endoscopist with extensive experience in EMR. Considering the total number of patients and resections performed in this study, it may be possible to see more accurate bleeding, perforation and stricture rates if the number of participants were to increase significantly and if endoscopists with variable range of experiences were to perform the procedure.

The visualisation through the Captivator cap is potentially better compared with the Duette cap. This is due to the position of the bands on the Captivator cap which are placed at the very proximal end of the cap, allowing a clear and unobstructed view through the transparent cap. This visualisation is further improved with each release of a rubber band. Improved view through the Captivator cap was based on the endoscopist’s experience with the devices and not formally assessed in our study. A formal analysis of visualisation through the Captivator cap was analysed by Schölvinck et al. that showed significantly higher overall median score for the visualisation with the Captivator cap [16]. The endoscopist also noted that the passage of accessories through the working channel of the scope was better with the Captivator device; however, this was not formally assessed, but again previously confirmed by Schölvinck et al. that showed the passage of accessories to be significantly easier with the Captivator device [16].

There are several limitations to our retrospective study. First, retrospective collection of data may have resulted in information bias and may have underestimated adverse events. Secondly, this study was performed by an experienced endoscopist at a high-volume tertiary referral centre with extensive experience in resection of large and complicated esophageal neoplastic lesions, which may have influenced the results significantly and therefore we may have observed different results if the procedure were performed by endoscopist with less experience. Third, EMR specimens were placed in formalin post resection, and then sent to the pathology lab for measurement of their dimensions and histological analysis. Formalin may have affected the size of these specimens and therefore the measured dimensions may have been under or overestimated. The specimens were not measured directly after the resection. Fourth, in order to create a pseudopolyp, the EMR cap was angulated against the esophageal wall and the mucosa was suctioned into the cap until a complete red out was visualised on the screen, prior to the deployment of the band. The quantity and volume of the suctioned mucosa into the cap is dependent on the angulation of the cap against the mucosa, where in the esophagus the resection may be taking place, and the elasticity of the tissue. The angulation of the cap against the mucosa was not controlled for in each group. In addition, tissue elasticity and fibrosis can affect the volume of mucosa suctioned into the cap. Variable prior exposure to acid reflux and scarring may have altered the tissue elasticity and fibrosis amongst some patients, limiting the volume of tissue being suctioned and subsequently affecting the size of the resected specimens. Fifth, device selection was done at the outset of each case, which was non-randomised and not controlled for in the study and at the endoscopist’s discretion. This introduces a selection bias. Finally, we measured the surface area of each EMR specimen by multiplying the minimum diameter by maximum diameter of each specimen. These two dimensions are not independent of each other and therefore the calculated surface area may have created an artificial endpoint.

In conclusion, our data show that both the Captivator and the Duette MBM Devices demonstrate excellent safety and efficacy to successfully resect delineated esophageal mucosal lesions in treatment naive patients with BE neoplasia. The Captivator device can resect larger specimens and therefore may be preferred for en bloc resections of larger complex esophageal lesions. This may improve procedure time by reducing the number of overall resections which would contribute to a reduction of total procedure time for piecemeal ER. Improved visualisation and passage of accessories through the working channel and comparable bleeding and perforation rates are features that are desirable by senior and trainee endoscopist. A large-scale randomised controlled trial to compare the two endoscopic devices in order to define efficacy and safety in more detail would confirm these findings further.
